# Suicide news on Peruvian television channels: evaluation of compliance with WHO’s reporting recommendations

**DOI:** 10.17843/rpmesp.2023.403.12776

**Published:** 2023-09-27

**Authors:** Kelly Broncano-Rivera, Carlos H. Contreras-Pizarro, Rubén Valle

**Affiliations:** 1 Faculty of Medicine, Universidad Nacional Mayor de San Marcos, Lima, Peru. Universidad Nacional Mayor de San Marcos Faculty of Medicine Universidad Nacional Mayor de San Marcos Lima Peru; 2 San Fernando Scientific Society, Lima, Peru. San Fernando Scientific Society Lima Peru; 3 Center for Research in Clinical Epidemiology and Evidence-Based Medicine, Research Institute, Faculty of Human Medicine, Universidad de San Martín de Porres, Lima, Peru. Universidad de San Martín de Porres Center for Research in Clinical Epidemiology and Evidence-Based Medicine, Research Institute Faculty of Human Medicine Universidad de San Martín de Porres Lima Peru

**Keywords:** Suicide, World Health Organization, Television, Communications Media, Mental Health, Peru

## Abstract

**Objective.:**

To determine if suicide news reports broadcasted by Peruvian television channels comply with the World Health Organization (WHO) recommendations for reporting suicide news.

**Materials and methods.:**

Quantitative and descriptive study. The unit of analysis was the suicide news broadcasted by eight Peruvian television channels during the years 2020 and 2021. News were classified into three categories: news about suicide, attempted suicide and suicide prevention. We used an evaluation instrument composed of WHO recommendations.

**Results.:**

A total of 126 news reports were analyzed; but none of these complied fully with the WHO recommendations. The news reports on suicide or attempted suicide complied with 4 of 13 recommendations. Most reports complied with avoiding to publish suicide notes (97.4%); on the other hand, educating about suicide and its prevention, and not disseminating myths was the recommendation least complied with (0.9%). Suicide prevention news reports complied with 5 of 7 recommendations.

**Conclusions.:**

Suicide news reports on Peruvian television in 2020 and 2021 showed low compliance with WHO recommendations. Communication professionals have a fundamental role in suicide prevention, limiting sensitive information and disseminating helpful information. It is essential for communicators to be aware of these recommendations and for the journalism team and mental health professionals to work together in the communication of news related to suicide.

## INTRODUCTION

Suicide is a serious public health problem, affecting all countries without exception. According to the World Health Organization (WHO), during the period from 2000 to 2019, suicide was the fourth leading cause of death among young people aged 15-29 years for both sexes ^1^. WHO reported 703,000 cases of suicide worldwide in 2019 ^1^. In South America, the age-adjusted suicide rate was 6.8 per 100 000 population between 2010 and 2014 ^2^. In Peru, the National Informatics System of Deaths reported 1267 cases of suicide during 2020 and 2021 ^3^. However, the actual number of cases could be higher due to possible errors in the registration of deaths ^4^.

Multiple factors at the individual, relational, community, societal and systemic levels have been associated with these events, which are closely related. For example, the loss of a job can directly influence a person’s well-being (individual-level factor), but this event, in turn, can be linked to an economic recession (societal-level factor) ^5^. Risk factors include previous suicide attempts, mental disorders, harmful use of alcohol and other substances, hopelessness, family history of suicide, genetic or developmental alterations of various neurobiological systems, chronic pain and illness, job or financial loss, lack of social support, trauma or abuse, stress, access to usable means of suicide, barriers in accessing health services, stigma associated with help-seeking behaviors, and inappropriate media reporting ^5^.

Inappropriate media coverage of suicide is also a social risk factor ^6^. Evidence shows that inadequate coverage of suicide can increase the risk of suicidal behavior in vulnerable people ^5^ (e.g., people with depression or previous suicide attempts), who when exposed to sensationalized news about suicide may commit suicide by imitation ^6^. How the impact of the media is related to subsequent suicide can be explained by social learning theory or identification theory. The former emphasizes that most human behaviors are learned by observation and that vulnerable people may learn from the media that suicide is a solution to problems. On the other hand, the identification theory points out that individuals tend to identify with people similar to themselves, so they develop a kind of attachment that encourages them to imitate suicidal behavior ^6^. As an example, we can mention what happened in Europe in the 18th century after the publication of Goethe’s novel in which a young man takes his own life after a love rejection ^6^. This same phenomenon occurred after the death of the actor Robin Williams in 2014, since, during the 5 months following the event, the suicide rate in the United States increased by 9.8 % above the baseline value ^7^.

The potential negative effects of inappropriate dissemination of suicide news in the media have led the WHO to issue guidelines for appropriate reporting ^8^^-^^10^. The WHO has published three documents as of 2017 that seek to reduce sensationalism, detailed description of the method, and location of the event in news coverage of suicide ^8^^-^^10^. In addition, they consider protective information such as helplines should be included ^10^. These guidelines are not mandatory, although their use by the media is strongly recommended ^10^. Thus, Spain, in the Clinical Practice Guide for the Prevention and Treatment of Suicidal Behavior, suggests adopting their use ^11^.

In Peru, the technical guidelines on suicide published in 2007 and 2022 by the Ministry of Health (MINSA) did not address the issue of the appropriate reporting of suicide news by the media ^12^^,^^13^. However, the recent publication of Law No. 31627 in November 2022 (Law that modifies Law No. 30947, Mental Health Law, in order to strengthen the prevention and promotion of mental health of children and adolescents and other vulnerable populations) introduced “Chapter X: Information for the prevention of suicide” in the Mental Health Law ^14^. That section states that any news reporting a case of suicide should include the message, “A suicide can be prevented if we talk about it in time. If you need advice, guidance or help, call toll-free 113, option 5. We are here to help you” ^14^. The objective with this message is to encourage the search for help and to channel it through the official means proposed by the Peruvian State. In addition, it is noted that this message can be modified to ensure its effectiveness and should be adapted according to the type of media (e.g. written, audiovisual, etc.) ^14^.

The scarce evidence from Peru shows that Peruvian media partially comply with WHO recommendations. For example, a study published in 2019 found that digital newspapers in Argentina and Spain were more compliant with expert recommendations on appropriate reporting of suicide news compared to two Peruvian newspapers ^15^. Given the relevance of appropriate reporting of suicide news in the media, the present study aims to determine compliance with WHO recommendations on the reporting of suicide news broadcasted by Peruvian television channels during the years 2020 and 2021. This type of media was chosen because it is the most widely consumed weekly (99%, according to a national survey), with news programs being the most preferred ^16^.

**KEY MESSAGES**

Motivation for the study. Appropriate communication of suicide events by the media is a well-known preventive measure.

Main findings. We found that no news report fully complied with WHO recommendations. The most complied with recommendation was to avoid publishing suicide notes left by the person, while the least complied with was to educate the population about suicide and its prevention, and not to spread myths.

Implications. The media should be trained and apply the WHO recommendations for the reporting of suicide news.

## MATERIALS AND METHODS

### Study design and population

A quantitative, descriptive study. The population consisted of suicide news broadcasted by eight Peruvian television channels during the years 2020 and 2021. Six open signal channels were selected: five of them considered to have the greatest national reach ^17^, and one was from the television channel of a radio broadcaster ^18^; and two cable television channels, selected for their specialization in news broadcasting ^18^.

*Selection criteria*

News related to confirmed suicide cases, suicide attempts and news about suicide prevention were included. News items referring to the same suicide case were also considered, given that these events are often covered by multiple television channels. In these cases, the inclusion of the news item depended on whether it was originally from the broadcasting channel and included the logo of the transmitting channel it (e.g., if a news item broadcast by channel A showed the logo of channel B in the video or image, that news item was excluded as it was not completely original to channel A).

On the other hand, news that dealt with topics such as suicide bombing, euthanasia and fictitious suicide (e.g., in movies) were excluded, as well as those that used the word “suicide” in a figurative sense (e.g., political suicide), those with inaccessible audiovisual material, which were part of complete recordings of newscasts (for feasibility reasons). In addition, news items with limited information on suicide were excluded; this occurred when suicide was a secondary topic, such as in main news stories about other events (e.g., a murder) or when the news item was too brief to be evaluated according to the 13 or 7 required items, which will be described later.

*Unit of analysis*

News about suicide: 1) news about suicide *per se*: news conveying the death of a person by a self-inflicted harmful behavior with the intent to die ^19^; 2) news about attempted suicide: news reporting potentially injurious non-fatal self-inflicted behaviors performed with the intent to die, and which may or may not result in injury. This group also included suicidal “threats” (a term not recommended by Crosby *et al*.) and suicide attempts interrupted by the same or another person ^19^; and 3) suicide prevention news: news focusing on epidemiology, warning signs, and means of help to prevent suicide, without providing information on individual or collective suicides. They seek to provide psychoeducation in the field of suicide.

### Variables and instrument

The evaluation instrument was comprised by the recommendations issued by WHO, considering the three documents published to date (2000, 2008 and 2017) ^8^^-^^10^. The synthesis, made by Acosta *et al*. (2017) and taken into account for this study, shows that there are 28 recommendations, and advises they should be used as a checklist ^20^. Our study only included recommendations that met three criteria: 1) contained examples or explanations in any of the three documents mentioned above, since this allowed an objective assessment; 2) recommendations that refer to elements external to the news report; and 3) recommendations described as subjective and not evaluable ^20^. Consequently, 13 items were analyzed for news items reporting suicide cases (Table 1). Since the prevention news did not provide information on individual or collective suicides, they were evaluated for only 7 of the 13 items (Table 1).

**Table 1:** WHO recommendations for the media to report suicide news

Recommendation: What not to do.	Type of news			
	Suicide/attempted suicide news		Suicide Prevention News	
	Is the criterion included?	Reason for exclusion	Is the criterion included?	Reason for exclusion
1. Do not sensationalize suicide.	No	Did not contain examples or explanations for their assessment.	No	Did not contain examples or explanations for their assessment.
2. Avoid using the word “suicide” in the headline, as well as stating the method or location of the suicide.	Yes	-	No	This type of news did not report on a suicide or attempted suicide.
3. Use caution when using photographs or videos. Do not post photographs or videos of the victim in a fatal state, of the method used, of the suicide scene, or dramatic photos (e.g., photos of people on ledges or similar, or of the instruments used).	Yes	-	Yes	-
4. Do not publish suicide notes in any of its possible forms (paper, text messages, messages on social networks or e-mail messages).	Yes	-	No	This type of news did not report on a suicide or attempted suicide.
5. Avoid placing the news in a prominent place. Relevant data should appear on inside pages.	No	Referred to external elements of the news report.	No	Referred to external elements of the news report.
6. Avoid reporting specific details or explicit description of the method used in the suicide or attempted suicide.	Yes	-	No	This type of news did not report on a suicide or attempted suicide.
7. Avoid providing detailed information about the location of the suicide or suicide attempt.	Yes	-	No	This type of news did not report on a suicide or attempted suicide.
8. Do not glorify the person who has died by suicide.	No	Did not contain examples or explanations for their assessment.	No	Did not contain examples or explanations for their assessment.
9. Do not present suicide as normal.	Yes	-	No	This type of news did not report on a suicide or attempted suicide.
10. Do not present suicide as a solution to problems or as a way to cope with them.	No	Did not contain examples or explanations for their assessment.	No	This type of news did not report on a suicide or attempted suicide.
11. Do not present suicide as a consequence of simplistic reasons.	Yes	-	Yes	-
12. Do not use religious or cultural stereotypes.	No	Did not contain examples or explanations for their assessment.	No	Did not contain examples or explanations for their assessment.
13. Do not expose suicidal behavior as an understandable response to social and cultural changes or devaluation.	No	Did not contain examples or explanations for their assessment.	No	Did not contain examples or explanations for their assessment.
14. Do not blame.	No	Did not contain examples or explanations for their assessment.	No	Did not contain examples or explanations for their assessment.
15. Avoid unwarranted repetition of news about suicide.	No	Referred to external elements of the news report.	No	Referred to external elements of the news report.
Recommendation: What to do.				
1. Refer to suicide as an event, not an achievement.	No	Did not contain examples or explanations for their assessment.	No	Referred to external elements of the news report.
2. Highlight alternatives to suicide, either through generic information or through individual stories that illustrate how to cope with adverse circumstances, suicidal ideation, and how to ask for help.	Yes	-	Yes	-
3. Provide information on community resources and help lines.	Yes	-	Yes	-
4. Provide information on risk factors and warning signs.	Yes	-	Yes	
5. Convey the frequent association between depression and suicidal behavior, and that depression is a treatable disorder.	Yes	-	Yes	
6. Offer a message of solidarity to survivors at a time of deep grief, and provide phone numbers for survivor support groups, if available.	Yes	-	No	This type of news did not report on a suicide or attempted suicide.
7. Take the opportunity to educate the public about the facts about suicide and suicide prevention, and do not spread myths about suicide.	Yes	-	Yes	
Other recommendations on suicide				
1. Authentic and reliable sources shall be used for statistics and shall be interpreted carefully and correctly.	No	Did not contain examples or explanations for their assessment.	No	Did not contain examples or explanations for their assessment.
2. Be especially cautious when reporting celebrity suicides.	No	Subjective and non-assessable recommendation	No	Subjective and non-assessable recommendation
3. Work closely with health authorities in the presentation of the facts.	No	Subjective and non-assessable recommendation	No	Subjective and non-assessable recommendation
4. Be careful with content, also in time-pressured situations.	No	Subjective and non-assessable recommendation	No	Subjective and non-assessable recommendation
5. Show due consideration for people who have lost a loved one (e.g., conducting an interview with a bereaved family member should be carefully considered, as they are in a vulnerable situation and are at increased suicidal risk).	No	Subjective and non-assessable recommendation	No	Subjective and non-assessable recommendation
6. Be aware that media professionals themselves may be affected by news stories about suicide.	No	Subjective and non-assessable recommendation	No	Subjective and non-assessable recommendation

The following information was also extracted from each news report: sex of the victim, place where the suicide/attempt occurred, time of transmission of the news report, method used, causes attributed to the event and whether the suicide occurred after a homicide or attempted homicide (Table 2).

**Table 2:** Characteristics of suicide news, period 2020-2021 (N=116).

Variable	N	%
Type of news		
Suicide	71	61.2
Attempted suicide	45	38.8
Transmission schedule		
Morning	62	53.4
Afternoon	16	13.8
Night	38	32.8
Sex of the victim		
Man	99	85.3
Woman	17	14.7
Method used		
Jumping from height	40	34.5
Use of firearms	39	33.6
Hanging	13	11.2
Use of a sharp weapon	8	6.9
Jumping to roads	4	3.4
Substance use	2	1.7
Starting a fire	1	0.9
Not mentioned	9	7.8
Causes attributed to the event		
Unique	73	62.9
Multiples*	12	10.4
Unknown	31	26.7
Associated to homicide or attempted homicide		
No	64	55.2
Yes	52	44.8

*Multiple: The news reported that the suicide was due to more than one cause (e.g., “the deceased was suffering from depression and was going through economic and labor problems”).

### Data collection

**Figure 1:**
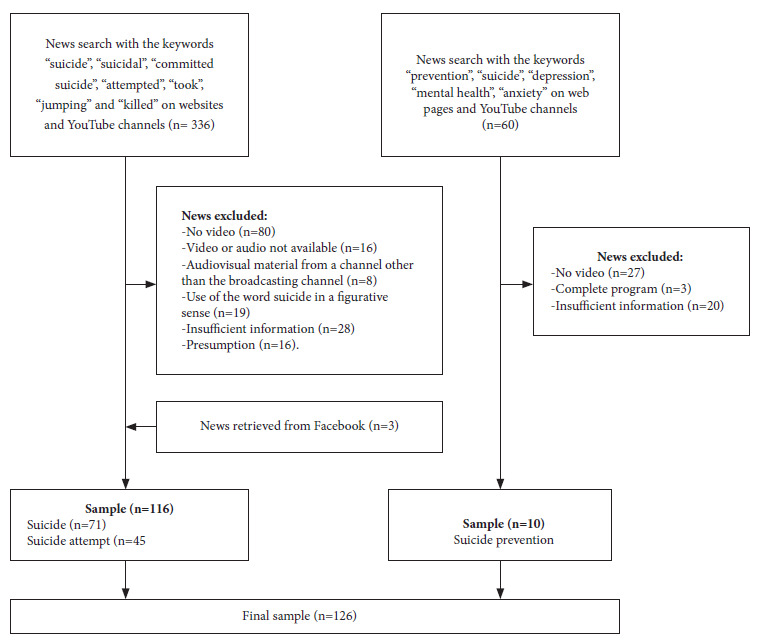
Flowchart for the selection of the news reports that made up the study.

A comprehensive search was conducted on the websites and Youtube accounts of each TV channel from March 15 to March 31, 2022. The selection flowchart is described in Figure 1. We carried out a complementary search on Facebook whenever a given case was not found on the websites and Youtube. The videos were viewed via Youtube or the website of the TV channels through successive meetings between two study evaluators, KBR and CHCP. At each meeting, each evaluator reviewed the video at least twice, took notes and subsequently collated their observations. When differences arose in the rating of an item in the instrument, the evaluators reviewed the video jointly to reach a consensus. This meant watching the video 2 to 3 times, with the aim of extracting audio fragments or screenshots from the video and backing up their arguments. In cases where immediate agreement was not reached, the news report was flagged and a subsequent discussion was scheduled, which usually took place 2 to 3 days later.

### Statistical analysis

An information extraction form was prepared in Microsoft Excel, in which each recommendation had two options (does comply/does not comply). The data were transferred to Stata version 14 (StataCorp, College Station, TX, USA), where they were evaluated using descriptive statistics. The number of recommendations that were followed by the news reports was evaluated using the median and interquartile range, while compliance with each recommendation was evaluated using absolute and relative frequencies.

### Ethical Aspects

The approval of an institutional Ethics Committee was not necessary because the data used in this study are available on freely accessible web platforms.

## RESULTS

A total of 126 suicide news reports broadcasted by television channels (six free-to-air and two cable) during 2020 and 2021 were retrieved. We found that 53.9% (n=68) of news items were broadcasted during 2021. The largest number (n=78) of news reports was obtained from the newscasts of two free-to-air television channels. Ten news items reported on suicide prevention and 116 on cases of suicide or attempted suicide.

*News about suicide or attempted suicide*

Seventy-one news reports on suicide cases and 45 on attempted suicide were transmitted on Peruvian television channels during years 2020 and 2021. Eighty-six news reports (74.1%) occurred in the cities of Lima and Callao, 62 news (53.4%) were broadcasted in morning hours and in 52 news reports (44.8%) the suicide/suicide attempt occurred after a homicide (Table 2). Jumping from height and the use of a firearm were the most frequently used methods. We found that 62.9% of the cases were attributed to single causes (e.g., suffering from bullying or cyberbullying, suffering from depression, avoiding police arrest, etc.) (Table 2).

All news reports met at least one recommendation, and two news reports met nine recommendations (1.7%), which was the highest number of recommendations met by a single report; the median compliance with WHO recommendations was 4 (interquartile range: 3-5). No news report met all 13 WHO recommendations. The recommendations that were mostly complied with were “avoid publishing suicide notes” (97.4%) and “avoid presenting suicide as normal” (95.7%), and the recommendations least complied with were “provide information on community resources and helplines” (0.9%) and “educate the population about suicide and its prevention, and do not spread myths” (0.9%) (Table 3).

**Table 3:** Compliance with 13 WHO recommendations on suicide news, period 2020-2021.

Recommendations	Suicide news (n=71)		Suicide attempt news (n=45)		Total (n=116)	
	n	%	n	%	n	%
Avoid publishing suicidal notes.	68	95.8	45	100	113	97.4
Avoid presenting suicide as something normal.	67	94.4	44	97.8	111	95.7
Avoid presenting suicide as a consequence of simplistic reasons.	32	45.1	16	35.6	48	41.4
Avoid revealing the person’s identity.	16	22.5	25	55.5	41	35.3
Avoid using the word “suicide”.	17	23.9	15	33.3	32	27.6
Avoid detailing the methods used.	23	32.4	4	8.9	27	23.3
Avoid the use of photographs or videos.	9	12.7	6	13.3	15	12.9
Mention association between depression and suicidal behavior.	6	8.5	6	13.3	12	10.3
Highlight alternatives to suicide with generic information or ways of coping.	2	2.8	6	13.3	8	6.8
Offer a message of solidarity to suicide survivors.	NA	NA	2	4.4	2	4.4
Provide information on risk factors and warning signs.	2	2.8	1	2.2	3	2.6
Provide information on community resources and helplines.	1	1.4	0	0	1	0.9
Educate the population about suicide and its prevention, and do not spread myths.	0	0	1	2.2	1	0.9

NA: Not assessable

*News on suicide prevention*

Ten news reports addressed the topic of suicide prevention on Peruvian television. The median compliance with WHO recommendations was 5 (interquartile range: 3.7-6). All news reports complied with at least three recommendations, with six being the highest number of recommendations complied with, found in four news items. No news report met all seven WHO recommendations. The recommendations that were mostly complied with were “avoid presenting suicide as a consequence of simplistic reasons” and “highlight alternatives to suicide with generic information or forms of coping”; and the least complied with was “mention association between depression and suicidal behavior” (Table 4).

**Table 4:** Compliance with WHO recommendations regarding the reporting of news on suicide prevention on Peruvian television channels, 2020-2021.

Recommendations	News (n=10)	%
Avoid presenting suicide as a consequence of simplistic reasons.	10	100
Highlight alternatives to suicide with generic information or ways of coping.	10	100
Provide information on risk factors and warning signs	8	80
Educate the population about suicide and its prevention, and do not spread myths.	6	60
Avoid the use of photographs or videos	5	50
Provide information on community resources and helplines	5	50
Mention association between depression and suicidal behavior	4	40

## DISCUSSION

This study evaluated compliance with WHO recommendations regarding the report of suicide news broadcasted on Peruvian television. It is noteworthy that no news report fully complied with the WHO recommendations and that, overall, news reports on attempted or completed suicide complied with 4 of the 13 recommendations and news items on suicide prevention complied with 5 of the 7 WHO recommendations. These results have implications for suicide prevention, since an appropriate reporting of news helps to reduce the potential imitative effect from the dissemination of a particular case, in addition to increasing the protective effect by providing useful information and counseling for people with suicidal thoughts ^10^.

Our results show that the news broadcasted by eight Peruvian television channels do not follow WHO recommendations on the reporting of suicide news. Inadequate reporting increases the risk of imitation related to the degree of identification of a person with the deceased. Imitation can be vertical, applied to people considered admirable or enviable; or horizontal, applied to people who are more socially similar, and in whom it is possible to recognize oneself or one’s own history ^6^. On the other hand, the appropriate reporting of this type of news has a protective role. Evidence indicates that appropriate communication could reduce suicidal ideation, increase life satisfaction, provide knowledge about suicide-related issues, and promote help-seeking behaviors ^10^.

There are some aspects to consider in the evaluation of the WHO recommendations on the broadcasting of suicide news. Several news reports included the word “suicide” in the headline or were mentioned by the communicators. In this regard, WHO notes that the phrase “committed suicide” implies criminality and increases stigma in those who have lost a person to suicide ^10^, so they recommend using terms such as “died by suicide” or “took his or her own life” ^10^. Although risk factors for suicide are multiple, the news reports explained suicide in relation to single causes (e.g., depression, cyberbullying, etc.). Instead, it is suggested to provide a comprehensive explanation about the potential factors involved which allows to increase awareness in the population ^10^. Many news reports published photographs or videos of the victim or the scene. Studies in the area indicate that these materials can trigger suicidal behavior in vulnerable people, for example, after a personal crisis ^10^. Therefore, WHO emphasizes the importance of using these materials with care, and suggests not to show the victim’s body or the suicidal act ^10^.

The methods used in suicide should also be omitted due to their negative effect on the audience. For example, after the reenactment of the suicide of the main character in the television series 13 Reasons Why, there was an increase in queries about suicide on the Google platform ^21^. We identified explicit narratives such as “he twirled the rope several times around his neck” or details that could have been omitted, such as the floor from which a person threw himself or herself. Because of the imitative behavior that may exist in suicide ^6^, we suggest including a warning message stating that the news report will address the topic of suicide ^14^. However, we did not identify reports that included warning messages before broadcast. Very few news reports about attempted suicide or suicide offered prevention information. This is not an isolated fact and is reported in several papers. For example, the study conducted by Ferreira *et al*. (2021) found a compliance rate of 5.6% with the item “provides information on risk factors and warning signs” ^22^. In the Netherlands, an analysis of 296 news items found that only one news report (0.3%) described coping behaviors other than suicide by an individual ^23^.

The news reports on suicide prevention showed higher rates of compliance with the recommendations due to the lower number of items with which they were evaluated. Although the news on suicide prevention debunked several myths on the subject, it should be noted that in one of the broadcasts it was implied that all forms of self-harm have suicidal intentions. This statement, mentioned by a health professional, may propagate unnecessary myths about suicide. In this regard, it should be noted that there is a group of self-injuries classified as non-suicidal, mostly present during adolescence, which can be a way of directing anger and frustration ^24^. In addition, the media should be responsible for the selection of their guests or the people to whom they rent television slots (within the same programs) to discuss suicide and mental health issues.

We identified that some news reports used the term suicide figuratively. In fact, we discarded 19 news stories of this (e.g., “political suicide” or “suicide bombing”). The use of a word that primarily carries a highly sensitive meaning outside of its “natural” context can desensitize the public to the issue, and increase the stigma that exists. A similar case has been previously reported with the misuse of the word psychosis ^25^. For example, a study conducted in a social network revealed that the word “psychosis” was used as equivalent to excessive fear and collective terror, and that it appears related to violent events, terrorist attacks or in contexts of political tension ^25^. Given the incorrect meaning imposed on psychosis by society, many countries in Asia refer to schizophrenia as integration disorder or attunement disorder ^26^ with the aim of reducing the stigma inappropriately assigned to this condition ^26^.

Communicators have a fundamental role in suicide prevention ^14^, limiting sensitive information and disseminating helpful information ^10^. To achieve this, knowledge of WHO guidelines is essential. However, evidence indicates that there is a lack of knowledge of these recommendations ^27^^-^^29^. Therefore, there is a need to include this information in the curricula ^27^. In this regard, the Pan American Health Organization has organized free training on responsible coverage of suicide for communicators in Latin America and the Caribbean ^30^^,^^31^. Despite these efforts, there is still debate among communicators as to whether and to what extent suicide cases should be reported ^27^. What is clear is that work on this topic should be developed in collaboration with mental health professionals to provide balanced and sensitive information ^10^^,^^32^.

The recent modification of the Mental Health Law (Law No. 30947) through the enactment of Law 31627 (2022) may allow for improvement in news coverage of suicide. However, it is necessary to monitor its compliance by all media outlets regardless of how the news is transmitted. We consider that media outlets could create an observatory, similar to that used by fact-checking teams ^33^, to corroborate compliance with the regulation. The observatory could operate on a web portal, where all news reports on suicide would be posted, allowing them to be evaluated and subsequently fed back for the implementation of improvements. The general population should also be attentive to the correct transmission of suicide news and if necessary, notify their complaint to the Radio and Television Advisory Council ^34^. Although not directly obliged by law, natural persons or health professionals who decide to publish suicide-related topics on their social networks can contribute to this effort by including help-seeking notes.

The present study has some limitations that should be considered. It is likely that some news items that did not include the word “suicide” in their headlines were not retrieved. Although the initial search algorithm used more keywords than those indicated in our flowchart, these were excluded from the final algorithm (words such as “life”, “hanged”, “immolate”, “immolated”, “shot”, etc.) because of their lack of specificity in identifying suicide-related news stories. Therefore, we consider the number of news items that exclude the term suicide in their headline (and are from the area) to be low. The population of news reports was reduced during the analysis due to the inaccessibility of eight news that initially met the inclusion criteria. We do not know the reasons behind the periodic removal of certain news reports from websites and YouTube channels, which should be considered in future work. Sixty-seven percent of news items were obtained from only two television channels; thus, a considerable part of the results is based on the transmission of these channels. In addition, television channels usually publish only a fraction of the news they broadcast on their web pages, so the analyzed news do not represent all the information reported at the time. Television channels tend to select news that they consider to be more relevant, so it is likely that some cases of suicide have not been covered when competing with other news that have a greater political, economic or social impact. Some WHO recommendations could not be evaluated due to the lack of specific measurement criteria. In spite of this, the items allow us to approximate the compliance of eight Peruvian television channels to the WHO recommendations. Finally, the non-publication of suicide notes was 96% in our study. However, the news reports did not clarify whether the victims had left any notes or whether they were collected as clues or evidence within the investigation process at the scene, which could have artificially influenced compliance with this criterion ^35^.

In conclusion, we found low compliance with WHO recommendations in news broadcasted by eight Peruvian television channels during the years 2020 and 2021. The most complied with recommendation was to avoid publishing suicide stories, while the least complied with was to educate the population about suicide and its prevention, and not to spread myths. The appropriate reporting of suicide news by the media can contribute to prevent the occurrence of suicide cases and encourage help-seeking. This requires close collaboration between communicators, the team in charge of preparing the news and mental health professionals.
