# Supplementary material for: Suicide news on Peruvian television channels: evaluation of compliance with WHO’s reporting recommendations
Source: Rev Peru Med Exp Salud Publica. 2023 Sep 27;40(3):307–16. doi: 10.17843/rpmesp.2023.403.12776 (PMC10953655; doi:10.17843/rpmesp.2023.403.12776)
Supplement: Translation

# Translation: Noticias de suicidio en canales de televisión peruanos: evaluación del cumplimiento de las recomendaciones de la OMS para su notificación

**Authors:** Kelly Broncano-Rivera, Carlos H. Contreras-Pizarro, Rubén Valle

**Affiliations:** 1 Facultad de Medicina, Universidad Nacional Mayor de San Marcos, Lima, Perú.; 2 Sociedad Científica de San Fernando, Lima, Perú.; 3 Centro de Investigación en Epidemiología Clínica y Medicina Basada en Evidencias, Instituto de Investigación, Facultad de Medicina Humana, Universidad de San Martín de Porres, Lima, Perú.

**Keywords:** Suicidio, Organización Mundial de la Salud, Televisión, Medios de Comunicación, Salud Mental, Perú

## Abstract

**Objetivo.:**

Determinar el cumplimiento a las recomendaciones de la Organización Mundial de la Salud (OMS) de las noticias sobre suicidio emitidas por canales de la televisión peruana.

**Materiales y métodos.:**

Estudio cuantitativo y descriptivo. La unidad de análisis fueron las noticias de suicidio emitidas por ocho canales de televisión peruana durante los años 2020 y 2021. Las noticias se clasificaron en tres categorías: noticias sobre suicidio, intento de suicidio y prevención del suicidio. Se utilizó un instrumento de evaluación compuesto por las recomendaciones de la OMS.

**Resultados.:**

Se analizaron un total de 126 noticias. Ninguna de estas noticias cumplió en su totalidad con las recomendaciones de la OMS. Las noticias sobre suicidio o intento de suicidio cumplieron con 4 de 13 recomendaciones, siendo la recomendación más acatada la de evitar publicar notas suicidas (97,4%), mientras que la menos cumplida fue la de instruir a la población sobre el suicidio y su prevención, y no difundir mitos (0,9%). Las noticias de prevención de suicidio cumplieron 5 de 7 recomendaciones.

**Conclusiones.:**

Las noticias sobre suicidio emitidas en la televisión peruana en los años 2020 y 2021 mostraron un bajo cumplimiento a las recomendaciones de la OMS. Los profesionales de la comunicación tienen un rol fundamental en la prevención del suicidio, limitando la información sensible y difundiendo información de ayuda. Es fundamental el conocimiento de estas recomendaciones por los comunicadores, y el trabajo conjunto entre el equipo periodístico y los profesionales de la salud mental en la comunicación de noticias relacionadas con el suicidio.

## INTRODUCCIÓN

El suicidio es un problema serio de salud pública, que afecta a todos los países sin distinción. De acuerdo con la Organización Mundial de la Salud (OMS), durante el periodo del 2000 al 2019, fue la cuarta causa de muerte entre jóvenes de 15 a 29 años para ambos sexos ^1^. La OMS reportó 703 000 casos de suicidio en el mundo en 2019 ^1^. En América del Sur, la tasa de suicidio ajustada por la edad fue de 6,8 por 100 000 habitantes entre 2010 y 2014 ^2^. En Perú, el Sistema Informático Nacional de Defunciones registró 1267 casos de suicidio durante los años 2020 y 2021 ^3^. Sin embargo, el número real de casos podría ser mayor debido a los errores que pueden existir en el registro de defunciones ^4^.

Múltiples factores a nivel individual, relacional, comunitario, social y sistémico se han asociado a estos eventos, los cuales se encuentran estrechamente relacionados. Por ejemplo, la pérdida de un trabajo puede influir directamente sobre el bienestar de una persona (factor a nivel individual), pero este evento, a su vez, puede estar vinculado con una recesión económica (factor a nivel social) ^5^. Entre los factores de riesgo se tiene al intento de suicidio anterior, trastornos mentales, consumo nocivo de alcohol y otras sustancias, desesperanza, antecedentes familiares de suicidio, alteraciones genéticas o del desarrollo de varios sistemas neurobiológicos, dolor crónico y enfermedades, pérdida de trabajo o financiera, falta de apoyo social, traumas o abuso, estrés, acceso a medios utilizables para suicidarse, barreras en acceso a los servicios de salud, estigma asociado con comportamientos de búsqueda de ayuda y la notificación inapropiada por los medios de comunicación ^5^.

La difusión inapropiada de noticias sobre suicidio en medios de comunicación es también un factor de riesgo social ^6^. La evidencia muestra que una inadecuada cobertura del suicidio puede aumentar el riesgo de conductas suicidas en personas vulnerables ^5^ (p.ej. personas con depresión o intento suicida previo), que al ser expuestas a noticias sensacionalistas sobre suicidio pueden cometer suicidio por imitación ^6^. La forma como se relaciona el impacto de los medios de comunicación con el suicidio posterior se puede explicar mediante la teoría del aprendizaje social o teoría de la identificación. La primera enfatiza que la mayoría de los comportamientos humanos se aprenden mediante observación y que personas vulnerables pueden aprender de los medios que una solución a los problemas es el suicidio. Por otro lado, la segunda señala que los individuos tienden a identificarse con personas similares a ellos, por lo cual desarrollan una especie de apego que los alienta a imitar la conducta suicida ^6^. Como ejemplo, se puede mencionar lo ocurrido en Europa en el siglo XVIII tras la publicación de la novela de Goethe en la que un joven se quita la vida tras un rechazo amoroso ^6^. Este mismo fenómeno ocurrió tras la muerte del actor Robin Williams en 2014, ya que, durante los 5 meses siguientes del evento, la tasa de suicidio en Estados Unidos aumentó en un 9,8 % por encima del valor de referencia ^7^.

Los potenciales efectos negativos de la difusión inadecuada de noticias sobre suicidio en medios de comunicación han llevado a que la OMS dicte pautas para su apropiada difusión ^8^^-^^10^. La OMS ha publicado tres documentos hasta el año 2017 que buscan reducir el sensacionalismo, la descripción detallada del método, y la ubicación del hecho en la cobertura de noticias de suicidio ^8^^-^^10^. Además, consideran incluir información de protección como líneas de ayuda ^10^. Dichas guías no son de uso obligatorio, aunque se recomienda fuertemente su uso por los medios de comunicación ^10^. Así, España, en la Guía de Práctica Clínica de Prevención y Tratamiento de la Conducta Suicida, sugiere adoptar su uso ^11^.

En el Perú, las guías técnicas sobre suicidio publicadas en 2007 y 2022 por el Ministerio de Salud (MINSA) no abordaron el tema de la apropiada difusión de noticias de suicidio por medios de comunicación ^12^^,^^13^. No obstante, la reciente publicación de la Ley N° 31627 en noviembre de 2022 (Ley que modifica la Ley N° 30947, Ley de Salud Mental, a fin de fortalecer la prevención y promoción de la salud mental de los niños, niñas y adolescentes y otras poblaciones vulnerables) introdujo el «Capítulo X: Información para la prevención del suicidio» en la Ley de Salud Mental ^14^. En ese apartado se indica que toda noticia que informe un caso de suicidio debe incluir el mensaje, «Un suicidio puede evitarse si lo hablamos a tiempo. Si necesitas consejo, orientación o ayuda, llama gratis al 113, opción 5. Aquí estamos para ayudarte» ^14^. El objetivo con este mensaje es alentar a la búsqueda de ayuda y canalizarla por los medios oficiales que el Estado Peruano propone. Además, se señala que dicho mensaje puede modificarse a fin de garantizar su eficacia y debe ser adaptado según el tipo de medio de comunicación (p.ej. escrito, audiovisual, etc.) ^14^.

La escasa evidencia que existe en el Perú muestra que los medios de comunicación peruanos cumplen parcialmente las recomendaciones de la OMS. Por ejemplo, un estudio publicado en 2019 encontró que los diarios digitales de Argentina y España seguían más las recomendaciones de los expertos sobre la comunicación apropiada de noticias de suicidio en comparación a dos diarios del Perú ^15^. Dada la relevancia que tiene el informar adecuadamente las noticias sobre suicidio en los medios de comunicación, el presente estudio tiene como objetivo determinar el cumplimiento de las recomendaciones de la OMS sobre la difusión de noticias de suicidio emitidas por canales de la televisión peruana durante los años 2020 y 2021. Se escogió este medio de comunicación por ser el de mayor consumo semanal (99%, según un sondeo nacional), siendo los noticieros los programas de mayor preferencia ^16^.

**MENSAJES CLAVE**

Motivación para realizar el estudio. Una de las medidas preventivas bien conocidas del suicidio es la apropiada comunicación de estos sucesos por los medios de comunicación.

Principales hallazgos. Se encontró que ninguna noticia cumplió completamente las recomendaciones de la OMS. La recomendación más acatada fue la de evitar publicar notas suicidas que dejó la persona, mientras que la menos cumplida fue la de instruir a la población sobre el suicidio y su prevención, y no difundir mitos.

Implicancias. Es necesario que los medios de comunicación se capaciten y apliquen las recomendaciones de la OMS para la difusión de noticias de suicidio.

## MATERIALES Y MÉTODOS

### Diseño y población de estudio

Estudio de enfoque cuantitativo, descriptivo. La población estuvo conformada por las noticias de suicidio emitidas por ocho canales de televisión peruana durante los años 2020 y 2021. Se seleccionaron seis canales de señal abierta: cinco de ellos considerados como los de mayor alcance a nivel nacional ^17^, y uno que provino del canal de televisión de una radiodifusora ^18^; y dos canales de televisión por cable, seleccionados por su especialización en la transmisión de noticias ^18^.

*Criterios de selección*

Se incluyeron noticias relacionadas con casos confirmados de suicidio, intentos de suicidio y noticias sobre prevención del suicidio. También se consideraron noticias que hacían referencia al mismo caso de suicidio, dado que estos eventos suelen ser cubiertos por múltiples canales de televisión. En estos casos, la inclusión de la noticia dependía de que está fuera originalmente del canal emisor y que incluyera el logo del canal que la transmitía (p. ej., si una noticia emitida por el canal A mostraba el logo del canal B en el video o la imagen, se excluía esa noticia al no ser completamente original del canal A).

Por otro lado, se excluyeron noticias que abordaron temas como el atentado suicida, la eutanasia y el suicidio ficticio (p. ej., en películas), así como aquellas que emplearon la palabra «suicidio» en sentido figurado (p. ej., suicidio político), aquellas con material audiovisual inaccesible, que formaban parte de grabaciones completas de noticieros (por razones de factibilidad del trabajo). Además, se excluyeron las noticias con información limitada sobre el suicidio. Esto ocurrió cuando el suicidio era un tema secundario, como en noticias principales sobre otros sucesos (p. ej., un asesinato) o cuando la noticia era demasiado breve para evaluarla según los 13 o 7 ítems necesarios, los cuales se describirán más adelante.

*Unidad de análisis*

Noticias sobre suicidio: 1) noticias sobre suicidio *per se*: noticia que transmite el deceso de una persona por una conducta dañina autoinfligida con intención de morir ^19^; 2) noticias sobre intento de suicidio: noticia que informa sobre conductas autoinfligidas potencialmente lesivas no mortales realizadas con la intención de morir, y que puede o no resultar en lesiones. En este grupo también se encontraban las «amenazas» suicidas (término no recomendado por Crosby *et al.*) e intentos de suicidio interrumpidos por la misma persona o por otra ^19^; y 3) noticias sobre prevención del suicidio: noticias que se enfocan en la epidemiología, señales de alerta y los medios de ayuda para prevenir el suicidio, sin proporcionar información sobre suicidios individuales o colectivos. Buscan brindar psicoeducación en el campo del suicidio.

### Variables e instrumento

El instrumento de evaluación estuvo constituido por las recomendaciones emitidas por la OMS. a partir de sus tres documentos publicados hasta la fecha (2000, 2008 y 2017) ^8^^-^^10^. La síntesis, realizada por Acosta *et al.* (2017) y que se tomó en cuenta para el estudio, muestra que existe un total de 28 recomendaciones, y recomienda su empleo para la investigación en forma de una lista de comprobación ^20^. Para el presente estudio, se incluyeron solo las recomendaciones que cumpliesen tres criterios: 1) contener ejemplos o explicaciones en alguno de los tres documentos antes mencionados, porque permitía una valoración objetiva; 2) recomendaciones que hagan referencia a elementos externos a la noticia; y 3) recomendaciones descritas como subjetivas y no evaluables ^20^. En consecuencia, se analizaron 13 ítems para las noticias que informaron sobre casos de suicidio (Tabla 1). Debido a que las noticias de prevención no brindaban información sobre suicidios individuales o colectivos, estas fueron evaluadas solo por 7 de los 13 ítems (Tabla 1).

**Tabla 1:** Recomendaciones de la OMS a los medios de comunicación para las noticias sobre suicidio. *(Continúa en la página 311)*

Recomendación: Qué no hacer	Tipo de noticia			
	Noticias de suicidio/intento		Noticias de prevención de suicidio	
	¿Criterio incluido?	Razón de exclusión	¿Criterio incluido?	Razón de exclusión
1. No trate el suicidio de forma sensacionalista	No	No contenía ejemplos o explicaciones para su valoración	No	No contenía ejemplos o explicaciones para su valoración
2. En el titular de la noticia evite el uso de la palabra «suicidio», así como la exposición del método o el lugar del suicidio	Sí	-	No	Este tipo de noticia no informaba sobre algún hecho de suicidio o intento
3. Sea prudente en el uso de fotografías o vídeos. No publique fotografías ni vídeos de la víctima en estado mortal, del método empleado, de la escena del suicidio, ni fotos dramáticas (por ejemplo, fotos de personas en cornisas o similares, o de los instrumentos utilizados).	Sí	-	Sí	-
4. No publique notas suicidas en ninguna de sus posibles modalidades (papel, mensajes de texto, mensajes en redes sociales o mensajes de correo electrónico).	Sí	-	No	Este tipo de noticia no informaba sobre algún hecho de suicidio o intento
5. Evite situar la noticia en un lugar destacado. Los datos relevantes deben figurar en páginas interiores.	No	Hace referencia a elementos externos de la noticia	No	Hace referencia a elementos externos de la noticia
6. Evite informar sobre detalles específicos o la descripción explícita del método usado en el suicidio o intento de suicidio	Sí	-	No	Este tipo de noticia no informaba sobre algún hecho de suicidio o intento
7. Evite aportar información detallada sobre el lugar del suicidio o intento de suicidio.	Sí	-	No	Este tipo de noticia no informaba sobre algún hecho de suicidio o intento
8. No glorifique a la persona que se ha suicidado.	No	No contenía ejemplos o explicaciones para su valoración	No	No contenía ejemplos o explicaciones para su valoración
9. No presente el suicidio como algo normal.	Sí	-	No	Este tipo de noticia no informaba sobre algún hecho de suicidio o intento
10. No presente el suicidio como una solución a los problemas, ni como un modo de afrontarlos.	No	No contenía ejemplos o explicaciones para su valoración	No	Este tipo de noticia no informaba sobre algún hecho de suicidio o intento
11. No presente el suicidio como consecuencia de razones simplistas.	Sí	-	Sí	-
12. No utilice estereotipos religiosos o culturales.	No	No contenía ejemplos o explicaciones para su valoración	No	No contenía ejemplos o explicaciones para su valoración
13. No exponga la conducta suicida como una respuesta comprensible ante los cambios sociales, culturales o la desvalorización.	No	No contenía ejemplos o explicaciones para su valoración	No	No contenía ejemplos o explicaciones para su valoración
14. No culpabilice.	No	No contenía ejemplos o explicaciones para su valoración	No	No contenía ejemplos o explicaciones para su valoración
15. Evite las repeticiones injustificadas de noticias sobre el suicidio.	No	Hace referencia a elementos externos de la noticia	No	Hace referencia a elementos externos de la noticia
Recomendación: Qué hacer				
1. Refiérase al suicidio como un hecho, no como un logro	No	No contenía ejemplos o explicaciones para su valoración	No	Hace referencia a elementos externos de la noticia
2. Resalte las alternativas al suicidio, ya sea mediante información genérica o mediante historias de personas, que ilustren cómo afrontar las circunstancias adversas, las ideas de suicidio, y cómo pedir ayuda	Sí	-	Sí	-
3. Proporcione información sobre recursos comunitarios y líneas de ayuda.	Sí	-	Sí	-
4. Aporte información sobre factores de riesgo y señales de alarma	Sí	-	Sí	-
5. Transmita la frecuente asociación entre la depresión y la conducta suicida, y que la depresión es un trastorno tratable	Sí	-	Sí	-
6. Ofrezca un mensaje de solidaridad a los sobrevivientes en momentos de profundo dolor, y proporcione números de teléfono de los grupos de apoyo para sobrevivientes, si los hay.	Sí	-	No	Este tipo de noticia no informaba sobre algún hecho de suicidio o intento
7. Aproveche la oportunidad para instruir a la población acerca de los hechos sobre el suicidio y su prevención, y no difunda los mitos sobre el suicidio	Sí	-	Sí	-
Otras recomendaciones				
1. Para las estadísticas deberán usarse fuentes auténticas y fiables e interpretarse cuidadosa y correctamente	No	No contenía ejemplos o explicaciones para su valoración	No	No contenía ejemplos o explicaciones para su valoración
2. Tenga especial cautela cuando informe sobre suicidios de personas famosas.	No	Recomendación subjetiva y no evaluable	No	Recomendación subjetiva y no evaluable
3. Trabaje estrechamente con las autoridades de la salud en la presentación de los hechos.	No	Recomendación subjetiva y no evaluable	No	Recomendación subjetiva y no evaluable
4. Sea cuidadoso en los contenidos, también en situaciones de presiones por tiempo	No	Recomendación subjetiva y no evaluable	No	Recomendación subjetiva y no evaluable
5. Muestre la debida consideración por las personas que han perdido un ser querido (por ejemplo, el realizar una entrevista a un familiar en duelo debe sopesarse cuidadosamente, ya que están en una situación de vulnerabilidad y tienen mayor riesgo suicida).	No	Recomendación subjetiva y no evaluable	No	Recomendación subjetiva y no evaluable
6. Sea consciente de que los propios profesionales de los medios de comunicación pueden verse afectados por las noticias sobre el suicidio.	No	Recomendación subjetiva y no evaluable	No	Recomendación subjetiva y no evaluable

Asimismo, se extrajo la siguiente información de cada noticia: sexo del agraviado, lugar donde ocurrió el hecho de suicidio/intento, hora de transmisión de la noticia, método empleado, causas atribuidas al hecho y si el suicidio ocurría posterior a un homicidio o intento de homicidio (Tabla 2).

**Tabla 2:** Características de las noticias de suicidio, periodo 2020-2021 (N=116).

Variable	N	%
Tipo de noticia		
Suicidio	71	61,2
Intento de suicidio	45	38,8
Horario de transmisión		
Mañana	62	53,4
Tarde	16	13,8
Noche	38	32,8
Sexo del agraviado		
Hombre	99	85,3
Mujer	17	14,7
Método empleado		
Lanzamiento desde altura	40	34,5
Uso de arma de fuego	39	33,6
Ahorcamiento	13	11,2
Uso de arma blanca	8	6,9
Lanzamiento a vías vehiculares	4	3,4
Uso de sustancias	2	1,7
Provocando un incendio	1	0,9
No menciona	9	7,8
Causas atribuidas al hecho		
Únicas	73	62,9
Múltiples^a^	12	10,4
Desconocidas	31	26,7
Asociado a homicidio o intento		
No	64	55,2
Sí	52	44,8

a Múltiples: En la noticia se informó que el suicidio se debía a más de una causa (por ejemplo, «el fallecido tenía un cuadro de depresión y atravesaba por dificultades económicas y laborales».

### Recolección de datos

Se realizó una búsqueda exhaustiva en las páginas web y Youtube de cada canal de televisión del 15 al 31 de marzo de 2022. El flujograma de selección se describe en la Figura 1. Cuando no se encontró un determinado caso en las páginas web y Youtube, se realizó una búsqueda complementaria en Facebook. Los videos fueron visualizados a través de Youtube o del portal web de los canales de televisión, a través de sucesivas reuniones entre dos evaluadores del estudio, KBR y CHCP. En cada reunión, cada uno revisó el video mínimamente dos veces, anotaron y posteriormente, cotejaron sus observaciones. Cuando surgían diferencias en la calificación de un ítem en el instrumento, los evaluadores revisaban el video de forma conjunta para llegar a un consenso. Esto implicaba ver el video de 2 a 3 veces, con el objetivo de extraer fragmentos de audio o capturas del video y respaldar sus argumentos. En casos en los que no se alcanzaba un acuerdo inmediato, se marcaba la noticia y se programaba una discusión posterior, que usualmente se realizaba de 2 a 3 días posteriores.

**Figura 2:** Flujograma de selección de las noticias que conformaron el estudio.

### Análisis estadístico

Se elaboró una ficha de extracción de la información en Microsoft Excel, en la que cada recomendación tenía dos opciones (sí cumple/no cumple). Los datos fueron transferidos al programa Stata versión 14 (StataCorp, College Station, TX, EE. UU.), donde fueron evaluados mediante estadística descriptiva. El número de recomendaciones acatadas por las noticias se evaluó con la mediana y rango intercuartílico, mientras que el cumplimiento de cada recomendación se evaluó mediante frecuencias absolutas y relativas.

### Aspectos éticos

No fue necesaria la aprobación de un Comité de Ética institucional debido a que los datos usados en el presente trabajo se encuentran disponibles en plataformas web de libre acceso.

## RESULTADOS

Se recuperó un total de 126 noticias de suicidio transmitidas por canales de televisión (seis de señal abierta y dos por cable) durante los años 2020 y 2021. El 53,9% (n = 68) de noticias se emitieron durante el 2021. La mayor cantidad (n = 78 noticias) se recuperó de los noticieros de dos canales de televisión abierta. Diez noticias informaron sobre prevención de suicidio y 116 sobre casos de suicidio o intento de suicidio.

*Noticias sobre suicidio o intento de suicidio*

Se identificaron 71 noticias sobre casos de suicidio y 45 sobre intento de suicidio trasmitidas en canales de televisión de Perú en los años 2020 y 2021. Ochenta y seis noticias (74,1%) ocurrieron en las ciudades de Lima y Callao, 62 noticias (53,4%) fueron transmitidas en el horario de la mañana y en 52 noticias (44,8%), el suicidio/intento de suicidio ocurrió posterior a un homicidio (Tabla 2). Sobre los métodos empleados, el lanzamiento desde altura y el empleo de arma de fuego fueron los más frecuentes. En relación con las causas, el 62,9% lo atribuyó a causas únicas (p. ej., sufrir de bullying o ciberbullying, padecer de depresión, evitar una detención policial, etc.) (Tabla 2).

Todas las noticias cumplieron al menos una recomendación, siendo nueve, el máximo número de recomendaciones cumplidas, que fue hallado en dos noticias (1,7%), mientras que la mediana de cumplimiento de las recomendaciones de la OMS fue 4 (rango intercuartílico: 3-5). Ninguna noticia cumplió las 13 recomendaciones de la OMS. Las recomendaciones más cumplidas fueron «evita la publicación de notas suicidas» (97,4%) y «evita presentar el suicidio como algo normal» (95,7%), y las recomendaciones menos seguidas fueron «proporciona información sobre recursos comunitarios y teléfonos de ayuda» (0,9%) e «instruye a la población sobre el suicidio y su prevención, y no difunde mitos» (0,9%) (Tabla 3).

**Tabla 3:** Cumplimiento a 13 recomendaciones de la OMS en las noticias de suicidio, periodo 2020-2021

Recomendaciones	Noticias de suicidio (n=71)		Noticias de intento suicida (n=45)		Total (n=116)	
	n	%	n	%	n	%
Evita la publicación de notas suicidas	68	95,8	45	100	113	97,4
Evita presentar el suicidio como algo normal	67	94,4	44	97,8	111	95,7
Evita presentar el suicidio como consecuencia de razones simplistas	32	45,1	16	35,6	48	41,4
Evita revelar la identidad de la persona	16	22,5	25	55,5	41	35,3
Evita el empleo de la palabra «suicidio»	17	23,9	15	33,3	32	27,6
Evita detallar los métodos empleados	23	32,4	4	8,9	27	23,3
Evita el uso de fotografías o videos	9	12,7	6	13,3	15	12,9
Menciona asociación entre depresión y conducta suicida	6	8,5	6	13,3	12	10,3
Resalta las alternativas al suicidio con información genérica o formas de afrontamiento	2	2,8	6	13,3	8	6,8
Ofrece un mensaje de solidaridad a los sobrevivientes de suicidio	NE	NE	2	4,4	2	4,4
Aporta información sobre factores de riesgo y señales de alarma	2	2,8	1	2,2	3	2,6
Proporciona información sobre recursos comunitarios y teléfonos de ayuda	1	1,4	0	0	1	0,9
Instruye a la población sobre el suicidio y su prevención, y no difunde mitos	0	0	1	2,2	1	0,9

NE: No evaluable

*Noticias sobre prevención de suicidio*

Se identificaron diez noticias que abordaron el tema de prevención de suicidio en televisión peruana. La mediana de cumplimiento de las recomendaciones de la OMS fue 5 (rango intercuartílico: 3,7-6). Todas las noticias cumplieron al menos tres recomendaciones, siendo seis el máximo número de recomendaciones cumplidas, observado en cuatro noticias. Ninguna noticia cumplió las siete recomendaciones de la OMS. Las recomendaciones más cumplidas fueron «evita presentar el suicidio como consecuencia de razones simplistas» y «resalta las alternativas al suicidio con información genérica o formas de afrontamiento»; y la recomendación menos seguida fue «menciona asociación entre depresión y conducta suicida» (Tabla 4).

**Tabla 4:** Cumplimiento a las recomendaciones de la OMS en la transmisión de noticias sobre prevención del suicidio en canales de televisión del Perú, 2020-2021.

Recomendaciones	Noticias (n=10)	%
Evita presentar el suicidio como consecuencia de razones simplistas	10	100
Resalta las alternativas al suicidio con información genérica o formas de afrontamiento	10	100
Aporta información sobre factores de riesgo y señales de alarma	8	80
Instruye a la población sobre el suicidio y su prevención, y no difunde mitos	6	60
Evita el uso de fotografías o videos	5	50
Proporciona información sobre recursos comunitarios y teléfonos de ayuda	5	50
Menciona asociación entre depresión y conducta suicida	4	40

## DISCUSIÓN

El presente estudio evaluó el cumplimiento de las recomendaciones de la OMS sobre la comunicación de noticias de suicidio transmitidas por televisión en el Perú. Resalta que ninguna noticia haya cumplido completamente las recomendaciones de la OMS y que, en general, las noticias de intento de suicidio o suicido consumado hayan cumplido 4 de las 13 recomendaciones y las noticias de prevención de suicidio 5 de las 7 recomendaciones de la OMS. Estos resultados tienen implicancias en la prevención del suicidio, puesto que una correcta transmisión de noticias ayuda a disminuir el potencial efecto imitativo a partir de la difusión de un determinado caso, además de incrementar el efecto protector al brindar información y consejería de utilidad para personas con deseos de cometer suicidio ^10^.

Los resultados encontrados indican que las noticias emitidas por ocho canales de televisión del Perú no siguen las recomendaciones de la OMS sobre la transmisión de noticias de suicidio. Una inadecuada transmisión aumenta el riesgo de imitación relacionado con el grado de identificación de una persona con la persona fallecida. La imitación puede ser vertical, aplicada a personas consideradas admirables o envidiables; u horizontal, aplicada a personas más parecidas socialmente, y en las que es posible reconocerse o reconocer la propia historia ^6^. Por otro lado, la transmisión adecuada de este tipo de noticias tiene un rol protector. La evidencia señala que una apropiada comunicación podría reducir las ideas suicidas, incrementar la satisfacción con la vida, brindar conocimiento sobre asuntos relacionados con el suicidio y promover comportamientos de búsqueda de ayuda ^10^.

Hay algunos aspectos que hay que considerar en la evaluación de las recomendaciones de la OMS sobre la emisión de noticias de suicidio. Varias noticias incluyeron la palabra «suicidio» en el titular o fue mencionada por los comunicadores. Al respecto, la OMS señala que la frase «se suicidó» implica criminalidad y aumenta el estigma en quienes han perdido a una persona por suicidio ^10^, por lo que recomiendan usar términos como «murió por suicidio» o «se quitó la vida» ^10^. Aunque los factores de riesgo para el suicidio son múltiples, las noticias identificadas explicaron el suicidio en relación con causas únicas (p.ej., depresión, ciberbullying, etc.). En su lugar, se sugiere brindar una explicación integral sobre los potenciales factores involucrados lo que permite incrementar la conciencia en la población ^10^. Muchas noticias publicaron fotografías o videos sobre la víctima o el lugar del hecho. Los estudios en el área indican que estos materiales pueden activar conductas suicidas en personas vulnerables, por ejemplo, después de una crisis personal ^10^. Por eso, la OMS enfatiza la importancia de emplear con cuidado estos materiales, y sugiere no mostrar el cuerpo de la víctima o el acto suicida ^10^.

Los métodos usados en el suicidio también deben ser omitidos por su efecto negativo en la audiencia. Por ejemplo, tras la recreación del suicidio del personaje principal de la serie *13 Reasons Why*, hubo un incremento de consultas sobre suicidio en la plataforma Google ^21^. En nuestro trabajo identificamos narraciones explícitas como «giró la soga varias veces alrededor de su cuello» o detalles que podrían haber sido omitidos, como el piso del cual se lanzó una persona. Por el comportamiento imitativo que puede existir en el suicidio ^6^, se sugiere incluir un mensaje de advertencia en el que se indique que la noticia va a abordar el tema del suicidio ^14^. No obstante, nosotros no identificamos noticias que incluyan mensajes de advertencia antes de la transmisión. Muy pocas noticias sobre intento de suicidio o suicidio ofrecieron información para la prevención. Esto no es un hecho aislado y se reporta en varios trabajos. Por ejemplo, el estudio conducido por Ferreira *et al.* (2021) encontró un cumplimiento del ítem «aporta información sobre factores de riesgo y señales de alarma» de 5,6% ^22^. En Países Bajos, un análisis de 296 noticias encontró que solo una noticia (0,3%) describió conductas de afrontamiento distintas al suicidio por un individuo ^23^.

Las noticias sobre prevención del suicidio presentaron mayores proporciones de cumplimiento de las recomendaciones por el menor número de ítems con que fueron evaluadas. Si bien las noticias sobre prevención de suicidio desmintieron varios mitos sobre el tema, debemos señalar que en una de las transmisiones se dio a entender que toda forma de autoagresión tiene intenciones suicidas. Esta afirmación, mencionada por un profesional de la salud, puede propagar mitos innecesarios respecto al suicidio. Al respecto, se debe recordar que existe un grupo de autolesiones catalogadas como no suicidas, presente mayormente durante la adolescencia y que puede ser una forma de dirigir la ira y frustración ^24^. Además, los medios de comunicación deben ser responsables en la selección de sus invitados o de las personas a quienes alquilan espacios televisivos (dentro de los mismos programas) para discutir temas de suicidio, y en general, temas de salud mental.

En el proceso de recuperación de noticias identificamos que algunas de ellas usaron el término suicidio de forma figurada. De hecho, nosotros descartamos 19 noticias de este tipo (p. ej., «suicidio político» o «bombardeo suicida»). El uso de una palabra que primariamente lleva un significado altamente sensible fuera de su contexto «natural» puede insensibilizar al público sobre el tema, e incrementar el estigma que existe. Un caso similar se ha observado con el mal empleo de la palabra psicosis ^25^. Por ejemplo, un trabajo realizado en una red social reveló que la palabra «psicosis» se empleaba como equivalente a miedo excesivo y terror colectivo, y que aparece relacionado a hechos violentos, atentados terroristas o en contextos de tensiones políticas ^25^. Dado el significado incorrecto impuesto a la psicosis por la sociedad, muchos países de Asia denominan a la esquizofrenia como trastorno de integración o trastorno de la sintonización ^26^ con el objetivo de disminuir el estigma inadecuadamente asignado a esta patología ^26^.

 Los comunicadores tienen un rol fundamental en la prevención del suicidio ^14^, limitando la información sensible y difundiendo la información de ayuda ^10^. Para lograrlo es fundamental el conocimiento de las directrices de la OMS. Sin embargo, la evidencia señala que existe un desconocimiento de estas recomendaciones ^27^^-^^29^. Por eso, existe la necesidad de incluir esta información en los planes de estudios ^27^. Al respecto, la Organización Panamericana de la Salud ha organizado capacitaciones gratuitas acerca de la cobertura responsable sobre suicidio dirigido a comunicadores de América Latina y el Caribe ^30^^,^^31^. A pesar de estos esfuerzos, existe aún debate por parte de los comunicadores sobre si se deben reportar los casos de suicidio y en qué medida ^27^. Lo que está claro, es que este trabajo debe desarrollarse en colaboración con profesionales de la salud mental para brindar una información equilibrada y sensible ^10^^,^^32^.

La reciente modificación de la Ley de Salud Mental (Ley N° 30947) mediante la promulgación de Ley 31627 (2022) puede permitir la mejora en la cobertura de noticias sobre suicidio. No obstante, es necesario supervisar su cumplimiento por todos los medios de comunicación independiente del medio de transmisión de las noticias. Nosotros consideramos que los medios de comunicación podrían crear un observatorio, similar al que emplean los equipos de *fact-checking*^33^, para corroborar el cumplimiento de la normativa. El observatorio funcionaría en un portal web, en el que se colocarían todas las noticias que informan sobre suicidio, permitiendo que sean evaluadas y posteriormente retroalimentadas para la implementación de mejoras. La población general debe también estar atenta a la correcta transmisión de noticias de suicidio y de ser necesario notificar su queja al Consejo Consultivo de Radio y Televisión ^34^. Aunque no están directamente obligadas por Ley, las personas naturales o profesionales de la salud que deciden publicar temas relacionados con el suicidio en sus redes sociales pueden contribuir a este esfuerzo incluyendo notas de búsqueda de ayuda.

El presente estudio tiene algunas limitaciones que se deben considerar. Es probable que algunas noticias que no incluyeron la palabra «suicidio» en sus titulares no hayan sido recuperadas. A pesar de que el algoritmo de búsqueda inicial usó más palabras claves que las indicadas en nuestro flujograma, estas se excluyeron en el algoritmo final (palabras como «vida», «ahorcó», «inmolarse», «inmoló», «disparó», etc.) debido a su falta de especificidad en la identificación de noticias relacionadas con el suicidio. Por lo tanto, consideramos que el número de noticias que excluyen el término suicidio en su titular (y que son del área) es bajo. La población de noticias se redujo durante el análisis debido a la inaccesibilidad de ocho noticias que inicialmente cumplían con los criterios de inclusión. Desconocemos los motivos detrás del retiro periódico de ciertas noticias en las páginas web y canales de YouTube, lo que debería considerarse en futuros trabajos. El 67% de noticias proviene de solo dos canales de televisión, por ende, una parte considerable de los resultados se basa en la transmisión de estos canales. Además, los canales de televisión suelen publicar solo una fracción de las noticias que transmiten en sus páginas web, por lo que las noticias analizadas no representan toda la información difundida en su momento. Los canales de televisión tienden a seleccionar noticias que consideran más relevantes, por lo que es probable que algunos casos de suicidio no hayan sido cubiertos al competir con otras noticias que poseen un mayor impacto político, económico o social. Algunas recomendaciones de la OMS no pudieron ser evaluadas debido a la falta de criterios específicos de medición. A pesar de esto, los ítems evaluados nos permiten aproximarnos a conocer la adherencia de ocho canales de televisión peruana a las recomendaciones de la OMS. Finalmente, la no publicación de notas suicidas fue de 96% en nuestro estudio. Sin embargo, las noticias analizadas no aclararon si las víctimas habían dejado alguna nota o si fueron recogidas como indicios o evidencias dentro del proceso de investigación en el lugar de los hechos, lo que podría haber influido artificialmente en el cumplimiento de este criterio ^35^.

En conclusión, encontramos un bajo cumplimiento de las recomendaciones de la OMS en las noticias transmitidas por ocho canales de televisión del Perú durante los años 2020 y 2021. La recomendación más acatada fue la de evitar publicar notas suicidas, mientras que la menos cumplida fue la de instruir a la población sobre el suicidio y su prevención, y no difundir mitos. La adecuada transmisión de noticias de suicidio por medios de comunicación puede contribuir a prevenir la ocurrencia de casos de suicidio y fomentar la búsqueda de ayuda. En ese trabajo se requiere la colaboración estrecha entre los comunicadores, el equipo encargado de elaborar la noticia y los profesionales de salud mental.
